# Toxicological

**Published:** 1863-02

**Authors:** 


*Toxicological.* It is suggested by M. TOSCANI that great care should be taken whenever salts of iron or mercury are being administered, not to give, contemporaneously, preparations containing hydrocyanic acid, (as for instance essence of bitter almonds,) since dangerous compounds are liable to be generated in such mixtures.

*Tannin* is recommended by PROF. KURZAK in cases of poisoning by *Strychnine.* Twenty or thirty times as much tannin should be exhibited. Vegetable acids and alcoholic drinks are to be avoided, on account of their increasing the solubility of the strychnia precipitated by the tannin.

DR. TSCHEPKE reports a case where a druggist's clerk took between eight and ten grains of Nit. of Strychnia, followed a little later by an addition of ten grs. Half an hour later having experienced no effects, he took ten grs. of Acet. of Morphia dissolved in an ounce of bitter almond water, and then lay down in bed. Ten minutes after he turned a quantity of Chloroform on his pillow to hasten his death. He lost consciousness for a short time, but soon revived, had convulsions several times, and itching of the nose and limbs.

Before taking the poisons, he had eaten freely of a sort of soup composed of cranberries and flour. Probably the tannin of the former partially neutralized the strychnia, whilst the farinaceous matters prevented absorption. The morphia may have proved to some extent antidotal.

Some spontaneous vomiting occurred, which was maintained actively by emetics of Ipecac and Antim. Two days afterwards no trace of the poisoning remained.

PROF. ANDERSON, of Glasgow, enumerates nine well determined substances obtained from Opium: Narcotine, morphine, codeine, paparerine, thebaine, narceine, meconine, meconic acid and theobslactic acid; besides three doubtful—pseudomorphine, porphyroxine and opianine.

